# Neurobiological foundations of multisensory integration in people with autism spectrum disorders: the role of the medial prefrontal cortex

**DOI:** 10.3389/fnhum.2014.00970

**Published:** 2014-12-04

**Authors:** Sonia Martínez-Sanchis

**Affiliations:** Department of Psychobiology, Faculty of Psychology, University of ValenciaValencia, Spain

**Keywords:** autism spectrum disorders (ASD), multisensory integration, medial prefrontal cortex (mPFC), default network, temporal multisensory binding

## Abstract

This review aims to relate the sensory processing problems in people with autism spectrum disorders (ASD), especially multisensory integration (MSI), to the role of the medial prefrontal cortex (mPFC) by exploring neuroanatomical findings; brain connectivity and Default Network (DN); global or locally directed attention; and temporal multisensory binding. The mPFC is part of the brain’s DN, which is deactivated when attention is focused on a particular task and activated on rest when spontaneous cognition emerges. In those with ASD, it is hypoactive and the higher the social impairment the greater the atypical activity. With an immature DN, cross-modal integration is impaired, resulting in a collection of disconnected fragments instead of a coherent global perception. The deficit in MSI may lie in the temporal synchronization of neural networks. The time interval in which the stimulation of one sensory channel could influence another would be higher, preventing integration in the typical shorter time range. Thus, the underconnectivity between distant brain areas would be involved in top-down information processes (relying on global integration of data from different sources) and would enhance low level perception processes such as over focused attention to sensory details.

## Introduction

In people with autism spectrum disorders (ASD), the existence of aberrant sensory perceptions may be as characteristic and disrupting as the presence of deficits in communication and social cognition (Hilton et al., 2007; O’Connor and Kirk, 2008; Donnellan et al., 2013). In line with the classification established by Miller et al. (2007), the sensory processing disorders (SPD) include disturbances in sensory modulation (SM) and alterations in the integration, organization and discrimination of sensory stimuli. Individuals with these disorders exhibit inappropriate responses to sensory inputs in such a way that the activities of daily life as well as emotional and behavioral patterns are severely affected. We can identify three types of SM disorders (SMD): hyper-responsiveness, hypo-responsiveness and sensory-seeking behavior. The hyper-responsiveness to sensory stimuli implies reactions disproportionately intense, rapid or prolonged. Hypo-responsiveness is unresponsiveness or slowness to respond to specific sensory stimulation. Finally, sensory-seeking behaviors encompass prolonged or intense sensory experiences reflecting craving/fascination with some stimuli.

The majority of people with autism present SMD in various sensory channels (Baranek et al., 2007; Tomchek and Dunn, 2007; Baker et al., 2008; Ben-Sasson et al., 2009; Lane et al., 2014). Regarding visual stimuli, some of them avoid bright lights and prefer the darkness; others are able to stare at intense light stimuli; and there are people who look lengthily at objects and people (Behrmann et al., 2006; Simmons et al., 2009). At vestibular level, many people within the autistic spectrum are hypo-responsive and seek this type of stimulation by spinning and rocking themselves (Lane et al., 2011). With regard to tactile stimulation, in some of these people hyper-responsiveness to certain stimuli (the shower, cutting hair or nails, being touched) could cohabit with hypoalgesia. Lastly, with respect to responsiveness to auditory stimuli, there have been cases of hyper-responsiveness to sounds that are not unpleasant for most people together with hypo-responsiveness to auditory linguistic stimuli (Ludlow et al., 2014).

In general, most information instead of being processed through one sensory modality arrives at the brain from multiple systems and favors a decision process about convergence or segregation of the different sensory inputs. Nevertheless, many people with ASD experience serious difficulties in multisensory integration (MSI), which results in an increment of their behavioral problems and an aggravation of the nuclear symptoms related to communication. Many subcortical regions such as the brainstem (superior colliculus), cerebellum and thalamus participate in this multisensory processing, although the superior temporal sulcus and prefrontal cortex (PFC) are also involved (Ghazanfar and Schroeder, 2006). The classical convergence model considers MSI as a feedforward process of unimodal signals when firing rate changes occur in neurons receiving convergent inputs from different sensory modalities. However, this approach does not explain sufficiently all aspects of MSI and a “temporal correlation hypothesis” has been proposed to account for a flexible setting for cross-modal interactions. It emphasizes the appearance of highly specific patterns of effective neuronal binding depending on synchronization of neural signals (“neural coherence”) (for a review, see Engel et al., 2012). Thus, MSI as a consequence of neural coherence could be associated with the modulatory role of the frontal areas in temporal patterns in cortical multisensory regions. In ASD, as discussed later, the abnormal cross-modal synchronization could be at the bottom of the impairment in MSI.

Different studies dealing with the neurobiological foundations of symptoms in ASD, including SPD, point out the important role played by the PFC. The orbital and medial prefrontal cortex (mPFC) is an area which is comprised of the medial wall (medial frontal gyrus and anterior cingulate cortex) and ventral surface of the frontal lobe. The whole region (including the lateral PFC) is involved in regulating planning, decision-making and solving problems in everyday situations (Executive function). The orbital network receives sensory inputs from different modalities and participates in integrating them. The mPFC network, basically projects viscero-motor outputs through its connections to the brainstem, although it also receives a few sensory inputs. Additionally, both networks are closely connected to the thalamus and the limbic system (amygdala, hippocampus, nucleus accumbens and central striatum). It has been hypothesized that this region contributes to evaluating external events and generating autonomic or somatic changes, which would enable the choice of the best behavioral option depending on the “somatic markers”. The mPFC and the anterior cingulate cortex would be involved in the evaluative component and in forming associations between sensory stimuli, responses and outcomes (Ongür and Price, 2000; Ridderinkhof et al., 2004; Bissonette et al., 2013).

The mPFC is also part of the brain’s Default Network (DN), which also includes the posterior cingulate, the inferior temporal lobe and the hippocampal formation. This network is deactivated when attention is focused on a particular task and activated on rest when spontaneous cognition emerges (“Internal mentation hypothesis”), although it also seems to play an important role monitoring the environment (“Sentinel hypothesis”). Thus, if this were the case, it would contribute to the formation of integrated internal representations of the environment and the self. According to the sentinel hypothesis, in some cases the DN activity correlates positively with sensory processing tasks. When this system implicated in global attention is hypoactive, as in Balint’s syndrome, just one visual object is perceived at a time, which impedes the understanding of the scene as a whole, which is just the experience narrated by several persons with ASD.

Following the hypothesis outlined by Shalom (2009), sensory processing in typically developing persons can occur at three levels: basic level, integrative level and higher-order level and networks including mPFC regulate integrative level. Sensory processing problems, especially MSI, in people with ASD could be related to the integrative role of the mPFC. The purpose of the present review was to explore this topic by exploring four issues: neuroanatomical findings; brain connectivity and DN; global or locally directed attention; and temporal multisensory binding.

## Neuroanatomical findings

Postmortem studies in people with ASD have revealed early postnatal brain overgrowth, especially an overabundance of neurons in the PFC, which correlated with enlarged head circumference in children under 3 years old, this being the most consistent neuroanatomical evidence in autism (Hazlett et al., 2005; Courchesne et al., 2011a,b). Subcortical areas connected to the PFC and related to sensory processing, such as the thalamus and cerebellum, exhibit functional and anatomical alterations too.

Nair et al. (2013) found reduced functional and anatomical connectivity between the thalamus and prefrontal, parieto-occipital, motor and somatosensory cortex in children and adolescents with ASD (9–17 years old). Conversely, there was a relative temporo-thalamic overconnectivity which was greater in the right hemisphere. The fronto-thalamic underconnectivity correlated with the severity of symptoms assessed with the Autism Diagnostic Observation Schedule (Lord et al., 1999). The thalamus is not only a relay station of sensory and motor information, but also seems to filter the flow of information to the cortex. This suggests its possible involvement in sensory symptoms of autism. In fact, several studies have reported reduced thalamic volume and glucose metabolism in persons with ASD (Haznedar et al., 2006; Tamura et al., 2010). Hardan et al. (2008) explored the presence of abnormalities in the thalamus comparing subjects with autism and controls and although no volumetric differences were observed between either group, metabolic differences were found. The group with autism showed lower levels of glutamate and N-acetylaspartate (a functional marker of neuroaxonal tissue), suggesting an imbalance in oxidative stress with consequent neurotoxicity. These metabolic results also correlate with sensory disturbances evaluated by the Sensory Profile Questionnaire (SPQ; Dunn, 1999).

There are strong associations between the cerebellum and the PFC, and abnormalities in both areas have been associated with the severity of symptoms (Carper and Courchesne, 2000; Kumar et al., 2010). Several studies reveal that the size of the cerebellum (cerebellum hemispheres and vermis lobes VI and VII) is significantly minor in the population with ASD when comparing with controls and there is also a reduced number of Purkinje cells (Bailey et al., 1998; Courchesne et al., 1988; Fatemi et al., 2002; Webb et al., 2009). The cerebellum participates in motor planning, which could be understood as the prediction of sensorial consequences of a motor act. This structure compares the predictions with the real consequences and learns to correct deviations. In this prediction, the cerebellar cortex would be in charge of rapid unconscious processes and, additionally, the parietal lobe would be responsible for the slow conscious ones (D’Angelo and Casali, 2013). Studies using animal models have found some empirical evidence of the existence of two pathways to connect cerebellum and PFC (Rogers et al., 2011). The first circuit involves the dentate nucleus, brainstem (reticulo-tegmental nuclei, pedunculopontine nuclei and ventral tegmental area) and finally the mPFC. The second is from the dentate nucleus via the thalamus (mediodorsal/ventrolateral nuclei).

## Brain connectivity and the Default Network (DN)

The identification of abnormal patterns of neural connectivity has proven to be one of the most promising explanatory approaches that permits one to unify cognitive theories (theory of the mind or central coherence theory), neurobiological findings and a neuropsychological perspective (Hughes, 2007). Neuroimaging methods have evidenced the presence of inter- and intra-hemispheric as well as cortico-subcortical underconnectivity in people with ASD. However, these findings coexist with data supporting just the opposite, i.e., the presence of hyper-connectivity using task-based and resting state functional connectivity (for review, see Vissers et al., 2012; Uddin et al., 2013). Functional connectivity has been studied by means of fMRI exploration of temporary associations between neurophysiological events using three approaches: regression analysis of a particular seed area; correlations analysis of multiple regions and; independent component analysis (ICA). In a systematic review, Uddin et al. (2013) indicate that the developmental perspective could contribute to conciliate these apparently contradictory results. These authors suggest that there is a shift from hyper- to hypo-connectivity in ASD with age, with two possible trajectories across adolescence: a reduced developmental increase or an abnormal pattern of functional connectivity. Coherent with the developmental perspective described above, stronger functional connectivity (ICA and seed-based) has also been found in the DN and other networks (salience, frontotemporal, motor and visual networks), predominantly in prepubertal children with ASD (Di Martino et al., 2011; Lynch et al., 2013; Uddin et al., 2013; Washington et al., 2014). There is also evidence, in adolescents and adults with ASD, of weak connectivity in long distance brain circuits such as reduced connectivity between DN nodes using correlations and ICA (Assaf et al., 2010; von dem Hagen et al., 2013; Tyszka et al., 2014).

The activity of mPFC and the posterior cingulate region, which are the midline portions of DN, correlates with internal mentation (self-referential thinking and theory-of-mind processes) and monitoring the environment for unexpected events. Spontaneous thoughts are absent and theory of mind impaired when mPFC is damaged (Mantini and Vanduffel, 2013). Although this network is intact in people with ASD, it is hypoactive in the mind’s resting state, in fact the higher the social impairment the greater the atypical activity during rest (Buckner et al., 2008). With a dysmaturation of the DN in ASD, the neural integration of signals from different sensory systems seems to be impaired, resulting in a collection of disconnected fragments instead of a coherent global perception. Additionally, different studies using evoked potentials have shown that discrimination of sounds of varying complexity (simple tones, complex tones and vowels) and the cortical representation are similar to those of the control in “oddball” sequences when attending to the stimuli. In this kind of task, the standard stimuli should be ignored while the novel stimulus (the “oddball”) must be attended to. On the contrary, processing is deficient when attention orienting is involuntary, especially if the stimulus is linguistic (Ceponiene et al., 2003; Dunn et al., 2008).

## Global or locally directed attention

Weak central coherence and enhanced perceptual functioning theories account for different hypotheses about perceptual processing in ASD people. In the former, it is suggested that there is an impairment in global processing, while in the latter it is proposed that the perception in ASD people is more locally oriented. Several studies using functional connectivity analyses have thrown light on the global and local level processing in people with ASD and their families (Briskman et al., 2001). One of the approaches consists of using local-to-global interference such as counting colored lines on a tridimensional object. Using this task, Liu et al. (2011) found that in the ASD group there was no global interference as revealed by the lower activation in executive brain areas and less synchronization between these regions and the visuospatial areas. When embedded figures tests or similar were used, the activity pattern in children, adolescents and adults with ASD was greater in right posterior regions (cuneus, occipital gyrus and inferior parietal areas). Finally, in visual matrix reasoning tasks (i.e., Raven matrices) greater occipital activation together with less prefrontal activity was observed in comparison with controls. Gadgil et al. (2013) explored differences in local and global level attention between adults with ASD and controls by means of a hierarchical, abstract shape recognition task. In the ASD group there was increased activation in the right PFC during the local condition, and increased activation of right occipital regions and decreased deactivation in mPFC during the global condition. The latter finding was consistent with less deactivation of the DN during global task processing in the ASD group, which could be related to greater distractibility under this attention condition. Additionally, the increased activation in occipital regions correlates with enhanced local level processing.

As has been hypothesized by Shalom (2005, 2009), perceptual problems in ASD could rely on processing in three levels: basic, integrative and logic, the integrative stage possibly being regulated by the mPFC. The visual recognition process implies the activation of both the mPFC and the anterior temporal cortex. The input from early visual areas first activates the mPFC when low spatial frequencies in the image predominate and globally oriented attention is required, then the temporal cortex region is activated. The mPFC selects the correct object representation and reward value assigned to it, and contributes to make the object itself more important than its sensory features when processed in the anterior temporal cortex. As indicated by fMRI studies, the superior temporal cortex is a neural center responsible for a wide range of high and low level MSI processes. Thus, as previously stated, one of the biological foundations of MSI problems could be the structural or functional abnormalities in the mPFC and superior temporal cortex (Stevenson et al., 2011; Mueller et al., 2013). Thus, relating data from connectivity and perceptual processing studies, the hypothesis of the modulating role of developmental factors is strengthened. The enhanced perceptual processing theory would explain functioning in the first years, while the weak central coherence approach could be more appropriate to clarify functioning later on.

## Temporal multisensory binding

The deficit in MSI, therefore, may lie in the temporal synchronization of neural networks, both local and distributed, since the ability to combine information from multiple sensory modalities to form a unified perception depends on the temporal synchrony of sensory stimuli. It has been hypothesized that frontal cortex could modulate temporal patterns in multisensory parietotemporal areas (Engel et al., 2012). Several studies have shown that the integration of low level visual and auditory stimuli is intact (Foss-Feig et al., 2010; Kwakye et al., 2011). There is, therefore, a certain degree of MSI when the audio-visual information is non-linguistic and simple, although there would be disruptions in temporal processing. Neural networks are intact, but atypical time intervals (temporal multisensory dysfunction) are needed to activate them. In the same way that the latency of response to sensory stimulation is longer, the time interval in which the stimulation of one sensory modality could influence another one in a different channel would necessarily be higher. Foss-Feig et al. (2010) evaluated this issue using the illusion “flash-beep”. In most subjects when pairing a visual (one flash) and several auditory stimuli (beeps) at the appropriate time interval, the illusion of perceiving two or more flashes was produced. The results of this study showed that this illusion also occurred in the group with autism, although the time window for the association of the two stimuli was larger. Using another kind of sensory tasks, these researchers also found similar results with respect to the existence of a wider temporal window MSI (Kwakye et al., 2011). However, if people with ASD are highly motivated this window could be smaller as has been hypothesized (Lawson, 2013).

Studies using electrophysiological and behavioral indicators (audio-visual reaction time task) of integration of audio-visual stimuli as well as judgment of audio-visual temporal order tasks have revealed deficits in general audiovisual temporal processing, and impairment in behavioral facilitation to multisensory inputs and in effective neural MSI (de Boer-Schellekens et al., 2013a,b; Brandwein et al., 2013; Collignon et al., 2013). These findings in multisensory temporal processing could be associated with the deficits in speech perception observed in people with ASD (Stevenson et al., 2014a,b). In fact, Foxe et al. (2013) found that high-functioning ASD children presented serious problems to integrate seen and heard speech especially as background noise increased. The developmental factor seems to be important since the impairment was ameliorated in adolescence.

## Summary

The insufficient connectivity between the mPFC and other areas distant from each other would be involved in top-down information processes relying on global integration of data from different sources (i.e., verbal and body language) and would enhance low level perception processes (bottom-up information) as in over focused attention to sensory details. The reduced deactivation in the mPFC and in the rest of the DN during global task processing together with a wider temporal window in MSI is consistent with the integrative modulatory role of mPFC as has been hypothesized. Researchers, people with ASD and their families have stressed the importance of understanding the degree to which sensory and movement anomalies in people with ASD can contribute to social impairment. In fact, many acts are mistakenly interpreted as non-compliance, reluctance, lack of interest and even aggressiveness when most of them are not volitional and are secondary to the idiosyncratic sensory and movement profile (Donnellan et al., 2013). Nevertheless, further investigation on the neurobiological basis of sensory symptoms and its relationship to other clinical features found in ADS is still needed to improve understanding and quality of life of persons with ASD and their families.

## Conflict of interest statement

The author declares that the research was conducted in the absence of any commercial or financial relationships that could be construed as a potential conflict of interest.
